# PreEpiSeizures: description and outcomes of physiological data acquisition using wearable devices during video-EEG monitoring in people with epilepsy

**DOI:** 10.3389/fphys.2023.1248899

**Published:** 2023-10-10

**Authors:** Mariana Abreu, Ana Sofia Carmo, Ana Rita Peralta, Francisca Sá, Hugo Plácido da Silva, Carla Bentes, Ana Luísa Fred

**Affiliations:** ^1^ Instituto de Telecomunicações, Lisboa, Portugal; ^2^ Departamento de Bioengenharia, Instituto Superior Técnico, Universidade de Lisboa, Lisboa, Portugal; ^3^ Lab EEG-Sono, Centro Hospitalar Universitário Lisboa Norte, Hospital de Santa Maria, Lisboa, Portugal; ^4^ Departamento Neurologia, Centro Hospitalar Lisboa Ocidental, Hospital Egas Moniz, Lisboa, Portugal; ^5^ Lisbon Unit for Learning and Intelligent Systems (LUMLIS), A Unit of the European Laboratory for Learning and Intelligent Systems (ELLIS), Lisboa, Portugal

**Keywords:** wearable devices, physiological signals, protocol design, epilepsy dataset, epilepsy monitoring, cardiorespiratory function, physiological data acquisition

## Abstract

The PreEpiSeizures project was created to better understand epilepsy and seizures through wearable technologies. The motivation was to capture physiological information related to epileptic seizures, besides Electroencephalography (EEG) during video-EEG monitorings. If other physiological signals have reliable information of epileptic seizures, unobtrusive wearable technology could be used to monitor epilepsy in daily life. The development of wearable solutions for epilepsy is limited by the nonexistence of datasets which could validate these solutions. Three different form factors were developed and deployed, and the signal quality was assessed for all acquired biosignals. The wearable data acquisition was performed during the video-EEG of patients with epilepsy. The results achieved so far include 59 patients from 2 hospitals totaling 2,721 h of wearable data and 348 seizures. Besides the wearable data, the Electrocardiogram of the hospital is also useable, totalling 5,838 h of hospital data. The quality ECG signals collected with the proposed wearable is equated with the hospital system, and all other biosignals also achieved state-of-the-art quality. During the data acquisition, 18 challenges were identified, and are presented alongside their possible solutions. Though this is an ongoing work, there were many lessons learned which could help to predict possible problems in wearable data collections and also contribute to the epilepsy community with new physiological information. This work contributes with original wearable data and results relevant to epilepsy research, and discusses relevant challenges that impact wearable health monitoring.

## 1 Introduction

Over the past years, research in epilepsy has been integrating more physiological information (i.e. biosignals) besides the electrical brain activity, captured by the Electroencephalography (EEG) (Ranjan et al., 2019). In fact, some devices with non-EEG biosignals have already been approved as medical devices by the Federal Drugs Administration (FDA), for their ability to detect of Generalised Tonic-clonic Seizures (GTCS) (Bruno et al., 2020). Examples include the Embrace Smartwatch by Empatica (McCarthy et al., 2016; Regalia et al., 2019), or the SPEAC device by Brain Sentinel (Whitmire et al., 2019). Additionally, non-EEG biosignals are also being used in epilepsy research for ambulatory daily monitoring of people with epilepsy (PWE), and for the development of seizure forecast and prediction models, where the most frequently mentioned device is the smartwatch E4 by Empatica (Meisel et al., 2020; Brinkmann et al., 2021; Vieluf et al., 2022). One essential aspect for the development of end-user solutions is the access to large datasets of multimodal quality data. This has been the focus of multiple international initiatives, such as the Remote Assessment of Disease and Relapse-Central Nervous System (RADAR-CNS), an initiative focused on ambulatory data collection is based on an open-source platform (RADAR-Base) to integrate data from different sources including smartphone and wearables (Ranjan et al., 2019).

The use of biosignals in epilepsy is especially relevant for the prevention of Sudden Unexpected Death in Epilepsy (SUDEP) (Van de Vel et al., 2013), which is thought to be a consequence of severe cardio-respiratory dysfunctions (Sivathamboo et al., 2020; 2021). With this in mind, our research group has given special focus to peripheral physiological signals which have shown to be of interest for these pathological changes, such as Electrocardiography (ECG), Respiration (RESP) and Chest Motion.

The PreEpiSeizures project was motivated by the collection of multimodal non-EEG data from PWE through wearables, in order to be leveraged towards the development of methods for early seizure detection, as well as expanding the understanding of epilepsy in different lenses. Although the use of biosignals in epilepsy is common, their contribution to different epilepsy types is still yet to prove, thus motivating the collection of comprehensive data to sustain and improve non-EEG based solutions. The rest of this document is structured as follows: Section 2 provides the current landscape of epilepsy-related data collection as well as commercial wearables which serve that purpose; Section 3 describes the experimental protocol employed in this project, along with the characteristics of the wearable devices used, together with the data quality assessment metrics; Section 4 details the main findings extracted from the data collected so far, including the analysis of the signal quality and patients’ metadata; Section 5 overviews several challenges faced in the context of wearable data collection, possible solutions, and presents a discussion of the results achieved. Lastly, Section 6 summarises the main outcomes and conclusions, followed by some future work directions for the PreEpiSeizures project.

## 2 Background

### 2.1 Datasets in epilepsy

Throughout the years, the epilepsy research community has been working in multi-centre collaborations. The joint efforts of stakeholders from different backgrounds, such as industry, healthcare and academia, as well as different countries, is essential to provide better and faster solutions to epilepsy. Some of the collaborations found were iEEG.org, SeizeIT, Epilepsy Ecosystem and EPILEPSIAE. Additionally, other publicly available datasets were found in the literature, which also comprise data from epileptic patients. The description of these datasets is summarised in Table 1 alongside the one proposed in this project. This includes the number of patients, their age, the modalities acquired, the number of seizures, the seizure types, the total duration and the setting.

**TABLE 1:** Comparison of public datasets with PreEpiSeizures, regarding number of patients, age, signal modalities, number of seizures, seizure types and total number of recording hours, and monitoring settings. If the number of seizures also includes subclinical, the number of subclinical seizures is specified in brackets. The numbers in brackets in the patients and total hours for the PreEpiSeizures are related to the wearable data, where the leftside numbers correspond to the hospital data. (* The 8 seizure types in the TUH EEG Corpus are: focal non specific seizure, generalised non-specific seizure, FAS, FIAS, absence, myoclonic, tonic and tonic-clonic seizures.)

Dataset name	# Patients	Age	Modalities	# Seizures	Seizure types	Total hours	Setting
PreEpiSeizures	59 (37)	[16,60]	ECG, Resp, ACC, EDA, PPG, EMG	348 (89)	FAS, FIAS, FUAS, FBTCS, E	5,838 (2,721)	Video-EEG
My Seizure Gauge Wearable (footnote 4)	27	-	EDA, PPG, ACC, EMG, GYR, EEG	226 (85)	GTCS, FIAS, FUAS, FAS, E	2640	Unclear
Siena Scalp EEG Detti (2020)	14	44 ± 14	EEG, ECG	47	FAS, FIAS and FBTCS	128	Hospital long-term
EPILEPSIAE Klatt et al. (2012)	30	41 ± 14	EEG, ECG	276	-	2881	Hospital long-term
NeuroVista Trial Cook et al. (2013)	15	44 ± 13	iEEG	2–12 monthly	Focal	>1y	Ambulatory
Bern-Barcelona Andrzejak et al. (2012)	5	-	EEG and iEEG	-	-	100 × 23.6s	Hospital
Bonn-Barcelona micro-, and macro- EEG database Martínez et al. (2020)	3	[20,39,47]	iEEG	0	-	66	Hospital long-term
TUH EEG Corpus Obeid and Picone (2016)	>10k	51 ± 56	EEG	≥3050	8 seizure types*	29.1*y*	Hospital long and short-term
New Delhi EEG Swami et al. (2016)	10	-	EEG	-	-	10 × 50 × 5.12s	Hospital
Epilepsy Seizure Recognition Andrzejak et al. (2001)	500	-	EEG	-	-	500 × 23.6s	Hospital short-term
CHB MIT Shoeb (2009)	23	10 ± 5.6	EEG	198	Not mentioned	969	Hospital long-term

The *iEEG.org* online platform^1^, supported by the National Institutes of Neurological Disorders and Stroke, provides access to over 900 datasets on human and animal epilepsy, as well as analytic and visualisation tools to aid data exploration. By inserting the corresponding keywords, the datasets are filtered to a desired EEG montage, population and seizure types, just to name a few. Nonetheless, the source of the signal is always assumed to be EEG and other physiological signals are not an option when filtering.

SeizeIT^2^ is another collaborative project within epilepsy, which is supported by the European Institute of Innovation and Technology (EIT Health). This consortium aims to tackle the continuous ambulatory monitoring of epilepsy, in order to accurately register seizures, thereby enabling the optimisation of treatment plans.

The Epilepsy Ecosystem^3^ (Kuhlmann et al., 2018) is a crowd-sourcing environment supported through the My Seizure Gauge project, supported by the Epilepsy Foundation of America, the Aikenhead Centre for Medical Discovery at St. Vincent’s Hospital Melbourne, the University of Melbourne, Monash University and Seer Medical. Its goal is to improve the performance of seizure prediction algorithms with the purpose of making them a viable option for PWE. They are responsible for gathering and maintaining two public datasets: NeuroVista Trial (Cook et al., 2013) and My Seizure Gauge Wearable^4^. Both datasets are available in the Seer platform requiring only a login setup. The NeuroVista trial (Cook et al., 2013) contains 15 patients that were submitted to an ambulatory intracranial EEG (iEEG) monitoring for more than 80 days. This dataset consists of a set of carefully selected patients whom experienced 2 to 12 monthly seizures, while still having a level of independence compatible with using the device for daily management. Another dataset made available in the Seer platform is the My Seizure Gauge Wearable, which is a collection of wearable data using 3 wearable devices: Empatica E4, Epilog (Frankel et al., 2021) and Byteflies (Swinnen et al., 2021). It comprises 27 patients that were monitored for approximately 4.4 ± 2.4 days. Although this dataset provides unprecedented wearable data in the context of epilepsy, it does not provide any information regarding the positioning of the devices nor some relevant clinical symptoms such as sleep/vigilance state and loss of awareness during the seizure events. Since no information was found regarding the acquisition conditions, we could not assume whether this dataset was acquired during video-EEG or in ambulatory.

The EPILEPSIAE database (Klatt et al., 2012) was created under a joint European project between Epilepsy Center, University Hospital of Freiburg; Center for Informatics and Systems, University of Coimbra; and Epilepsy Unit, CHU Pitie-Salpetriere, Paris. This database contains more than 275 patients that performed Video-EEG monitoring, focusing on the EEG signal. Still, since the ECG is also acquired by the hospital system, it is also present in this database. Several works have used this database both for EEG and ECG analysis (Truong et al., 2019; Gómez et al., 2020; Leal et al., 2021). However, EPILEPSIAE only makes available 30 patients for external researchers, which is always the same subset, without disclosing the type of seizures, or the clinical semiology. Moreover, it does not provide wearable data, only hospital data.

The Siena Scalp EEG Epilepsy Database (Detti et al., 2020) contains the data of 14 patients that performed the video-EEG monitoring in the Unit of Neurology and Neurophysiology of the University of Siena. This dataset is curated to the seizure episodes, hence for some patients, only the peri-ictal period is available to the public. The signal sources present in this database include EEG and ECG, both acquired by the hospital system.

Other epilepsy datasets also available in the literature were included in Table 1 for dimensionality comparison, however, they are out of the scope of this research, since they provide neither non-EEG data or wearable data. For more information in EEG datasets (Wong et al., 2023), provides a comprehensive review.

The majority of available datasets is focused on EEG analysis, and only a few also include the ECG signal acquired by the hospital system. Regarding the acquisition settings, four datasets contain data from long-term or video-EEG monitorings, and four datasets contain short-term EEG acquisitions. The EEG was intracranial in three datasets (indicated with iEEG), and the NeuroVista Trial is the only ambulatory dataset, to the best of our knowledge. The only multimodal wearable dataset found was My Seizure Gauge Wearable data, however it does not provide clear information on seizure types, the acquisition conditions, or the clinical manifestations of seizures. Most events annotated in this dataset have little seizure information, and for some of them it is unclear if they are even seizures or not. Larger datasets are needed to sustain the use of non-EEG data as lens for epileptic activity. Recently, projects such as My Seizure Gauge and Radar-CNS started to create multicenter datasets with hundreds of patients acquired in ambulatory scenarios (Böttcher et al., 2022). However, it was not yet proven the ability of these devices to capture pathophysiological manifestations in non-EEG modalities related to non-convulsive seizures. Hence more evidence based on data collected in a semi-controlled scenario (e.g. as the video-EEG monitoring), is essential towards the use of non-EEG physiological modalities in the prediction and forecast of epileptic seizures, and in the detection of non-convulsive epileptic seizures.

### 2.2 Peripheral multimodal devices

The ever-decreasing size of devices with built-in sensors, internal battery and powerful processors, allow continuous wearable long-term monitoring of movement patterns and physiological variables. A wearable device similar to a smartwatch can be decomposed in four main layers: top cover, electronics board, lithium battery and bottom cover^5^. The top cover serves as a protective shield for the other components; the electronics board contains sensors, communication antenna (Bluetooth or WiFi), memory, and MCU; the lithium battery provides a power supply; finally the bottom cover often contains the interface with the patient when necessary (e.g., dry electrodes). This example of a wearable device unfolds the physical constraint associated with each layer: on the one hand, multiple embedded sensors lead to more information, and larger battery sizes lead to longer continuous acquisitions; on the other hand, devices should be small and unobtrusive. Thus, there is a trade-off between the device’s size and its modus operandi.

The devices that can simultaneously record data from multiple sensors are more interesting for epilepsy monitoring since they can increase the chances of capturing manifestations from different seizure types, thus enhancing the performance of detection or early prediction algorithms (Verdru and Van Paesschen, 2020). Some devices are commercially available for the purpose of physiological signal acquisition, which could be used for research purposes. In Table 2, relevant devices found in the literature are described, which were validated and can record multimodal physiological signals. In this table, the devices are compared based on their target use, validation, modalities available, body location, battery duration, sampling frequency (FS) and data accessibility. For comparison, the bottom part of Table 2, contains the characteristics of the devices used in this project.

**TABLE 2:** Devices with multiple sensors, characterised with respect to their applicability to research, validation status, form factor or body positioning, battery duration, method to access the data, measured signals and sampling frequency.

Product	Research use	Validation	Modalities	Site	Battery	FS(Hz)	Data accessibility
EQ02^9^	Ambulatory Orphanidou and Drobnjak (2017), Athletes Kenny et al. (2017)	With Holter Akintola et al. (2016)	ECG, Resp, Temp, ACC	Vest	48 h	256	BT transmission
E4 footnote 5	Migraines Koskimäki et al. (2017), Stress Ollander et al. (2016)	CE Regalia et al. (2019)	EDA, PPG, Temp, ACC	Bracelet	+32 h	128	BLE to Cloud Storage
VisiMobile^10^	Ambulatory Weenk et al. (2017)	FDA	ECG, Resp, SpO_2_, Temp	Wristband	+12 h	-	WiFi transmission
Bioharness3 Johnstone et al. (2012)	Sports	HR validation Nepi et al. (2016)	ECG, Resp, Temp, ACC	Chest Strap	12–24 h	-	BLE transmission
Hexoskin^11^	Remote	With gold-standard Smith et al. (2019)	ECG, Resp, ACC	Smart Shirt	12 h	256	Cloud Storage or BLE
Vivosmart4^12^	Stress	With gold-standard Tedesco et al. (2019)	Optical HRV, SpO_2_, Light, ACC	Smartwatch	5 days	-	BLE transmission
Byteflies^13^	Epilepsy	CE	EEG, ACC, Resp, HR	Behind Ear	24 h	250	Cloud Storage
ArmBIT + ForearmBIT			ECG, EDA, EMG, PPG, ACC	Arm + Wrist	12 h	1,000	BT transmission
WristBIT			EDA, PPG, ACC	Wrist	24 h	1,000	BT transmission
ChestBIT			ECG, PZT, ACC	Chest	24 h	1,000	BLE transmission

9 https://equivital.com/

10 https://soteradigitalhealth.com/

11 https://www.hexoskin.com/

12 https://www.garmin.com/en-US/p/605739

13 https://byteflies.com/

Table 2 illustrates the characteristics that prove to be the most relevant in wearable devices applied to epilepsy. However, it also uncovers some limitations of current options: from restrictive form factors, to short battery life, lack of validation, limited storage options, or insufficient physiological information. In fact, this last point accurately illustrates one pressing issue in epilepsy research: the extent of the applicability of seizure detection devices is inherently related to their location/signals they acquire, for example, an ACC- or EMG-based device will not be able to detect non-motor seizures. Even though the research community’s intention is in detecting all seizure types, there is an imbalance, in both research and evidence, towards an overrepresentation of convulsive motor seizures (Bruno et al., 2020). Therefore, research efforts should be endorsed for other seizure types. Moreover, additional research is needed towards the applicability of multimodal wearables in epilepsy in ambulatory scenarios, especially combined with seizure risk forecasting.

The benchmark device for epilepsy research is the Empatica E4 smartwatch, since it is discreet, it captures four biosignals and its battery lasts for 32 h. However, the data management is performed in a cloud-based platform, which compromises our privacy protection clauses. Moreover, (Vandecasteele et al., 2017), have reported the higher reliability of ECG, comparatively to PPG, to obtain the heart rate and heart rate variability, which are considered significant autonomic change predictors. These two factors motivated the development of our own devices, designed to ensure the privacy preservation and the recording of relevant physiological modalities, such as the ECG, PZT, PPG, EDA and ACC (for chest motion).

## 3 Methodology

Data collection within the PreEpiSeizures project started as a collaboration from Instituto Superior Técnico (IST) with the EEG-Sleep Lab of Hospital de Santa Maria - Centro Hospitalar Universitário Lisboa Norte (CHULN), that is responsible for supervising video-EEG monitorings. This project was approved in 2015 by the ethics committee of CHULN. This collaboration was later extended to Centro de Epilepsia Refratária of Hospital Egas Moniz—Centro Hospitalar Lisboa Ocidental towards continuous monitoring in the scope of the PreEpiSeizures project underwent multiple iterations with the purpose of achieving a friendlier and more autonomous solution for patients. The approach is divided into the development of the wearable device and in the acquisition system for data collection and storage.

### 3.1 Wearable devices

The devices developed in this scope are based on BITalino (Da Silva et al., 2014), which allowed for the experimentation around form-factors and sensors modalities. The BITalino device consists of a core block with a microprocessor (MCU) and a Bluetooth (BT) module for communication, sensors for each physiological modality, and a lithium-ion polymer (LiPo) battery. BITalino has the ability of recording simultaneously up to 6 analog channels (with a FS up to 1000Hz). When all channels are being acquired, the first four channels also allow for 10-bit resolution, while the last two only record with 6-bit resolution.

**FIGURE 1:**
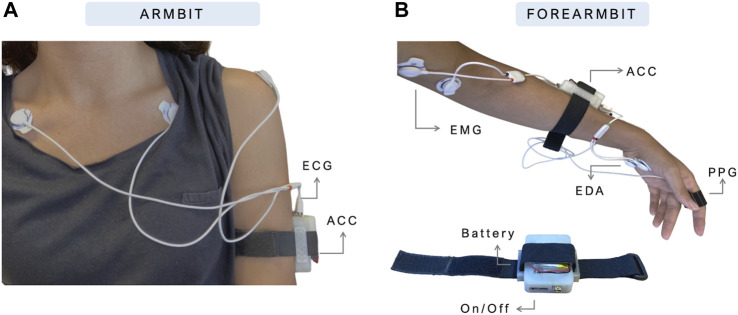
Prototypes of ArmBIT **(A)** and ForearmBIT **(B)**, the first wearable devices used to record multimodal data during video-EEG acquisitions.

The first prototypes developed within the PreEpiSeizures project were ArmBIT and ForearmBIT (shown in Figure 1), both consisting of one BITalino core block (with MCU and BT module), and respective sensors, all enclosed within a 3D printed case with an adjustable strap. The ArmBIT, which is displayed in left side of Figure 1, collected the equivalent of lead I ECG and the triaxial ACC of the upper arm. The ForearmBIT, represented in right side of Figure 1, was design to record the bicep’s EMG, EDA on the palm of the hand, fingertip’s PPG, and forearm’s triaxial ACC. Interface with the patient was performed with the use of pre-gelled Ag/AgCl electrodes. These two devices were used simultaneously and were connected by a cable, to enable synchronisation (not shown in Figure 1). Although this montage is acceptable for short-term data acquisitions, it becomes impractical for longer sessions. This is due to the prolonged use of pre-gelled electrodes and connection cables, which can cause discomfort, associated with the fact that wearables are secured to the arm, inhibiting patients from adopting certain sleeping positions. Since these two devices are both on the arm and physically connected by a cable, the remainder of this work uses ArmBIT to refer to the ensemble with both devices.

Given the higher resolution in the first four channels, these were usually used for the more relevant physiological signals (e.g., ECG, EEG, EMG and PPG). Since the ACC possesses three axis, and the human motion has frequencies lower than 5 Hz (Korohoda et al., 2013), two of its axis were usually acquired in the channels with lower resolution.

**FIGURE 2:**
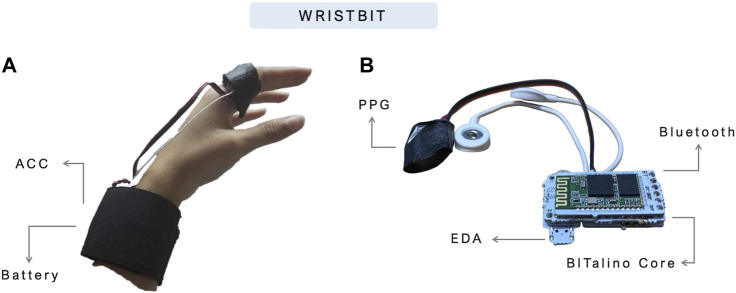
WristBIT prototype used in the PreEpiSeizures project. In the **(A)**, the WristBIT is being worn in the left hand, where the EDA and ACC are recorded in the wrist and the PPG in the finger. The **(B)** shows the interior of the wristband, with the BITalino main unit attached to the Bluetooth module and sensors.

The second iteration of the PreEpiSeizures wearable devices, consisted on rejecting gel electrodes and finding other ways to collect the same signals. The ForearmBIT was replaced by a BITalino assembled in a wristband (WristBIT), whereas the ArmBIT was replaced by a BITalino mounted in a chestband (ChestBIT). The WristBIT device (shown in Figure 2), is composed of an elastic wristband with a pocket to enclose the BITalino core block, as well as EDA and ACC sensors. The EDA signal was being recorded on the wrist, using two metallic electrodes encapsulated in the strechable fabric. Additionally, the finger strap enclosing the PPG sensor was connected to the wristband.

**FIGURE 3:**
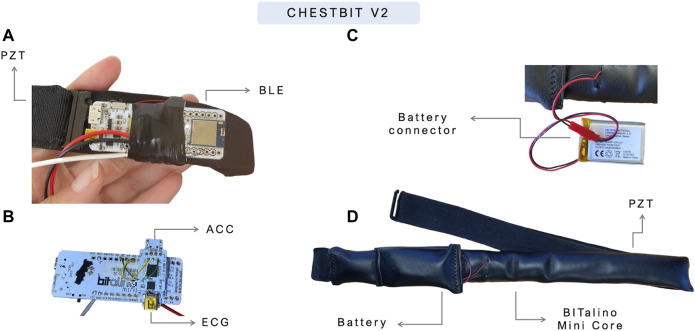
Current prototype of ChestBIT in the PreEpiSeizures project. On the **(A,B)** the ChestBIT components are shown, namely, the BItalino mini, and the mounted sensors ECG and ACC. On the **(C)** the connection between the battery and the BITalino Mini is shown, and in the **(D)**, the components are covered by a comfortable textile.

The ChestBIT is shown in Figure 3, and it was previously applied in other contexts within the research group (Manso et al., 2018). The chestband used was a Polar chest strap^6^, with the ECG sensor connected to the metal buttons of the chest strap. The BITalino MCU was glued to the chest strap, alongside the ACC, the Bluetooth module, and a piezoelectric PZT sensor used to record the RESP signal. The entire electronics was protected with a soft sponge and covered with a synthetic leather textile, to improve endurance. The battery remained outside, in a pocket, and a small opening allowed the battery connector to reach the BITalino power module; this enabled easier natter charging and replacement to ensure near-continuous operation. The on/off button was accessible through the side, since the left side of the textile was not fully closed.

To ensure greater battery life, the batteries used in ArmBIT and ForearmBIT were LiPo with 750 mAh and lasted for 12 h, which required a bi-diary battery exchange. However, the new wearables ChestBIT and WristBIT reduce the physical constrain of battery size, reason for which LiPo batteries of 1,600 mAh were used, requiring a single daily battery change.

### 3.2 Data quality assessment

The quality of all data recorded was assessed both for the hospital system and for the wearable devices, following the approach found in (Böttcher et al., 2022) for data quality evaluation. In this approach, the quality of the acquired data is assessed globally for each dataset, through statistical metrics (mean, median, standard deviation, minimum value and maximum value). These metrics are applied to the patient data variables, namely: duration, data completeness, and each biosignal quality.

The duration variable consists on the time span from the first timestamp marking the beginning of the acquisition until the last timestamp. It is presented in hours, minutes and seconds (H:M:S). The percentage of usable data (i.e., not loss) is measured by the data completeness. This variable is the ratio between the recorded duration and aforementioned duration variable. The recorded duration is given by all the timestamps associated to a data value between the first and last data points.

The ECG quality was evaluated in segments of 10 s and in three levels: low, medium and high. Low quality corresponds to an unusable segment, whether due to the presence of an artifact, or due to the loss of connection with the body. Medium quality (ECG MQ) corresponds to a segment where it is possible to extract rhythm information, in particular the heart rate and heart rate variability. In this scenario, the R peak detection is reliable, but the rest of the ECG waveform can be indistinguishable. Lastly, the high quality (ECG HQ) corresponds to a reliable and consistent depiction of the ECG wave throughout the segment. This qualitative stratification was also described in previous works and gives a perception of possible applications of the acquired data. To reach these levels, each segment is processed according to the following method: the R peaks locations are found and the instantaneous heart rate is calculated. If the heart rate is between 40 and 200 beats per minute, the low quality level is discarded (the low quality is chosen otherwise). Then, the ECG wave cycles are cross-correlated through Pearson correlation (between 0 and 1). If the cross correlation is above 0.8, then the segment is classified as high quality (and medium quality if below the threshold).

For the ACC, (Nasseri et al., 2020), evaluated the quality of Empatica E4 in the context of video EEG-monitoring, proposing as SQI the ratio between human motion activity frequency and the entire frequency range, as given by Eq. 1, where *p* refers to the power’s spectrum in the indicated frequency band and FN is the Nyquist frequency (half of the sampling frequency). In this paper, the metric was calculated for 4-s non-overlapping segments and averaged across 10-min intervals.

$${SQI}_{ACC}=\frac{P_{\left[0.5,8\right]}}{P_{\left[0.8,F_{N}\right]}}$$ (1)

The signal quality analysis of the RESP signal was calculated following the metric proposed by (Charlton et al., 2021) for a respiratory signal acquired through impedance pneumography. This metric consists on the analysis of valid breaths from segments with 32 s of length. For each segment, the peaks are extracted and used to find breath cycles. The segment is valid if the breaths respect some predefined conditions proposed by the authors. This metric results in a binary separation between high quality and low quality.

The EDA quality was retrieved from (Böttcher et al., 2022), where the binary decision high/low quality is based on two factors. The first is the signal amplitude, which has to be above 0.05 *μS*; then, the signal amplitude change rate is calculated as the ratio between the min-max amplitude and the first of these two extremes to appear. This rate should be below 0.2 in the assessment of EDA for windows with 2 s.

The aforementioned work, evaluates the quality of the PPG also in a binary low/high quality distinction, using the spectral entropy in windows of 4 s. A spectral entropy value should be below 0.8 for the segment to be considered high quality.

The decision to follow the quality assessment approaches taken by (Nasseri et al., 2020; Böttcher et al., 2022) enabled the comparison between our wearable devices and other datasets and data acquired in similar conditions. Both these works acquired wearable data using the Empatica E4.

### 3.3 Experimental protocol

When a patient is admitted to the hospital stay, they sign an informed consent from the hospital so that their data can be analysed and used for research purposes. After the hospital system setup, the patient is approached to participate in the wearable data collection as well. If accepted, a new informed consent is given for the use of wearable devices during the hospital stay. The proposed acquisition system collects data unobtrusively and only requires a daily short-time interruption for battery replacement. This section describes the process of accessing the wearable and the hospital data, the structure of the dataset, as well as the several formats in which each data source was saved.

#### 3.3.1 Hospital data extraction

The hospital data of the video-EEG monitoring consisted on the patient’s reports and hospital admission notes, along with the data recorded by the monitoring unit. The physiological data is always reviewed by an EEG technician and a neurologist resulting in a video-EEG monitoring report with all the relevant events annotated. This is usually saved as Word and PDF documents. The reports were only consulted with the purpose of retrieving the seizures’ onsets and details, being dissociated from the biosignals’ records, which are anonymised thus preserving patients’ and data privacy. The physiological data is stored in the hospital servers and accessed through a secure computer in the hospital’s facility. All physiological data files are retrieved with all acquired channels, varying from 24 (22 EEG channels and 2 ECG channels) up to 77 (72 EEG channels, 2 ECG, 1 SpO2, 1 EMG, and 1 EOG). However, most of the time, EOG and SpO2 are disconnected, and EMG is only connected in some motor seizures, usually on the deltoid or tibia.

Concerning the data extraction, we came across different systems in the two hospitals included in this study, which led to different extraction processes and file formats. The HSM video-EEG data is managed by the Nihon Kohden DMS system^7^; all HSM files are extracted in the European Data Format (EDF), resulting in files with 2 h of data, sampled at 1,000 Hz. Through the mne python package, it is also possible to use the raw Nihon Kohden files (.EEG format) (Gramfort et al., 2013). The HEM video-EEG data is managed by Micromed software^8^. All HEM files are extracted in the micromed raw file format (TRC), resulting in files with 2 h of data, sampled at 256 Hz.

In both hospital systems, two ECG channels are recorded, corresponding to the positive and negative channels. The difference between these channels results in the approximation of the Lead I derivation, sometimes requiring inversion of the signal (i.e., when the positive and negative electrodes are placed on the wrong side).

#### 3.3.2 Wearable data extraction

The acquisition setup relied on a processing unit to receive BITalino data in real-time and store it appropriately. This was initially performed using a laptop and Python script, but was later replaced by a more user-friendly acquisition system developed within our research group, named EpiBOX (Carmo et al., 2022). Nonetheless, both systems stored the data in the same structure.

The data collected by the wearables was saved every time the system disconnected, or after 1 h in a stabilised connection. These files are in the TXT format, comprising the data of one or two devices (if they are acquired simultaneously), with a list-like header (with one dictionary per device, where the key corresponds to the device MAC address) on the first few rows and the values over the next rows, in a table-like structure. All the header fields are detailed with examples in the Supplementary Material. Besides the header, each subsequent row corresponds to a new time instant (1/*FS*), where FS corresponds to the sampling frequency of 1,000 Hz. Each TXT file is named after its starting time (e.g., 2021-04-02 18-09-05.TXT). The table produced with two devices is equal to one device with the only difference being the addition of the second device’s columns. The order of first and second is known by the order in which they appear in the header. When retrieving data from the second device, it is necessary to recognise the presence of the first, and use its number of columns as an offset. This structure is easily scalable to additional devices.

## 4 Results

The results detailed in this section are divided into the signal quality analysis and the characteristics of the dataset acquired so far.

### 4.1 PreEpiSeizures data quality

The quality of biosignals was studied for all data acquired by the hospital system, the ChestBIT, the WristBIT and the ArmBIT and these results are summarised in Table 3. The hospital data was acquired for 59 patients totalling almost 6 k hours of ECG data. Data completeness is fairly high with a median of 99.21%. The majority of the signal was classified, on average, as high quality (around 70%), however, on average, 15% of the data was of poor quality. Regarding the ChestBIT, 37 patients used this device, with some sessions being more successful than others (this is observable by the minimum and maximum metrics). The data completeness is lower (in mean and median) when compared to the hospital system, which is expected since the system communicates through Bluetooth, hence being more susceptible to connection losses. Nonetheless, the discrepancy between the mean and the median indicates that the majority of acquisitions was mostly successful in terms of data completeness. In ChestBIT, more segments were discarded as poor quality, however, the median of low quality segments is also around 15% (100 - (ECG HQ + ECG MQ)). Interestingly, the median percentage of ECG HQ is higher in the ChestBIT than in the hospital system, and very few segments were classified as medium quality. The quality of RESP signals is on average 60%, which is reasonable.

**TABLE 3:** Signal quality analysis for the data acquired by the hospital system and the devices ChestBIT and WristBIT. The N indicates the number of patients and the total duration is in H:M:S. The first column indicates the statistical metric, followed by the data completeness (in %), the quality of the biosignals, where the ECG is divided in high and medium quality (in %) and the acquisition duration (in H:M:S).

Hospital data, *N* = 59				Total duration: 5,838:19:12
*p*	Data Completeness (%)	ECG HQ (%)	ECG MQ (%)	Duration
Mean	92.33	68.58	17.15	98:57:16
Median	99.21	71.83	16.11	94:33:40
SD	15.09	20.29	11.33	33:51:50
Min	43.40	4.10	0.88	10:43
Max	100	96.60	52.23	165:47:15

WristBIT, *N* = 9				Total Duration: 510:04:07	
*p*	Data Completeness (%)	EDA (%)	BVP (%)	ACC	Duration
Mean	70.64	92.84	64.33	0.44	56:39:46
Median	69.95	98.23	71.72	0.39	60:33:25
SD	21.11	13.73	25.31	0.19	15:21:54
Min	31.82	57.55	20.39	0.22	26:31:28
Max	93.38	99.86	89.72	0.80	76:25:50

ChestBIT, *N* = 37					Total Duration: 2,211:53:29	
*p*	Data Completeness (%)	ECG HQ (%)	ECG MQ (%)	PZT (%)	ACC	Duration
Mean	69.64	67.33	4.44	58.52	0.35	59:46:51
Median	85.98	82.03	3.02	61.63	0.34	61:20:44
SD	31.84	34.59	6.07	21.16	0.22	34:18:16
Min	1.15	0.0	0.0	1.81	0.0	10:20
Max	100	98.65	30.89	89.47	0.82	192:38:58

ArmBIT, *N* = 4					Total Duration: 156:14:54	
*p*	Data Completeness (%)	ECG HQ (%)	ECG MQ (%)	EDA (%)	BVP (%)	Duration
Mean	60.09	8.18*10^–3^	0.84	15.50	58.52	39:03:43
Median	59.19	0.0	0.64	2.03*10^–2^	61.63	7:27:19
SD	28.56	1.64*10^–2^	0.97	30.97	21.16	67:27:35
Min	26.35	0.0	0.0	0.0	1.81	1:26
Max	95.65	3.27*10^–2^	2.05	61.95	89.47	139:54:13

The WristBIT was only acquired in 9 patients, still, it comprises 500 h of data, with data completeness percentage comparable to the ChestBIT. The quality of the EDA signal was significantly high, however the PPG quality was significantly lower, since on average, only half of the segments have high quality. This was expected, since the PPG is highly susceptible to noise. Moreover, this is comparable to the results reported by (Böttcher et al., 2022) with the Empatica E4, where the mean and median quality of PPG data was in the range [51.5, 63.3]% for the datasets acquired in hospital settings. The *SQI*_*ACC*_ values of the WristBIT are in agreement with those reported by the study of (Nasseri et al., 2020).

### 4.2 PreEpiSeizures dataset

The total number of patients in this dataset is 59. From this cohort, 36 performed the video-EEG in Hospital de Santa Maria (HSM), while 23 performed the video-EEG in Hospital Egas Moniz (HEM). This dataset includes biosignals recorded by our wearable devices, as well as records from the hospital. The classification of seizure types follows the guidelines of the 2017 ILAE’s instruction manual (Fisher et al., 2017a; b). Focal seizures were classified as FUAS, whenever the awareness was not registered or tested. The ictal semiology described in this dataset, follows the 2022 ILAE’s glossary of terms (Beniczky et al., 2022).

#### 4.2.1 Hospital de Santa Maria

**FIGURE 4:**
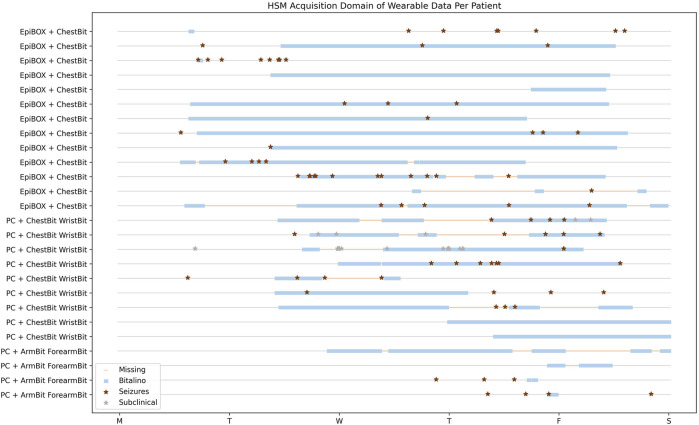
HSM Data Acquisition Temporal Overview. Patients with wearable and hospital data. The horizontal axis represents the temporal domain, from the beginning of the video-EEG monitoring, until its end. Each row consists in a single patient.

Figure 4 provides a visual depiction of the wearable data acquired so far in HSM. The horizontal axis represents the days of the week, whereas the vertical axis represents the different patients enrolled. The label of vertical axis contain the information of the wearable device and the acquisition system. The blue lines represent the wearable data, while the pink shade corresponds to the missing data. The red and grey diamonds represent clinical and subclinical seizures, respectively.

The first 4 patients enrolled in this study used the wearables ArmBIT and ForearmBIT during their video-EEG monitoring: in Figure 4, these are represented in the four bottom rows. These acquisitions lasted for 5 days and 19 h, 13 h, and 1 h and 30 min for the remaining two, respectively. However, only one of these acquisitions captured a seizure event (the patient with the longest acquisition did not experienced any seizure during their hospital stay, whilst the other patients’ seizures occurred outside the wearables’ recorded time spans). There were 10 patients that used the ChestBIT and WristBIT during their video-EEG monitoring, recording over 102 seizures. Their distribution according to seizure type is: subclinical = 63; FBTCS = 8; FAS = 5; FIAS = 17, and unknown focus = 9. The percentage of recorded data by these wearable devices was on average 53% ± 18% of the entire video-EEG monitoring stay (4 full days = 96 h). The number of actual recorded seizures by the wearable device is 19, from a total of 39 clinical seizures, which occurred during the monitorings.

The EpiBOX deployment as the acquisition system was mostly successful, with only 4 out of 13 patients having less than a day recorded (their recorded duration was: 13 h; 3 h; 12 min and 9 min). The ChestBIT enabled the recording of 30 out of 60 clinical seizures. Besides the mentioned patients so far, there was the case of patients who were enrolled in the study and only have hospital data. Since the hospital monitoring equipment provides ECG recordings, they were still included in the dataset, despite the lack of wearable data. 9 patients are included in this group with a total of 47 clinical seizures, with a distribution in seizure types of FBTCS = 6; FAS = 19; FIAS = 16 and subclinical = 6.

#### 4.2.2 Hospital Egas Moniz

**FIGURE 5:**
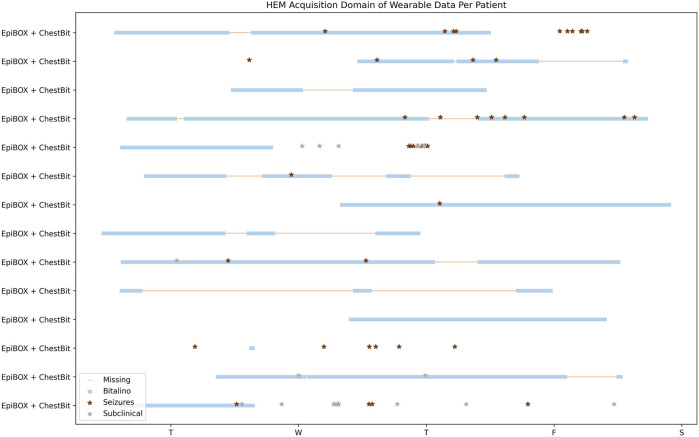
HEM Data Acquisition Temporal Overview. Patients with wearable and hospital data. The *X*-axis represents the temporal domain, from the beginning of the video-EEG monitoring, until its end. Each row consists in a single patient.

**FIGURE 6:**
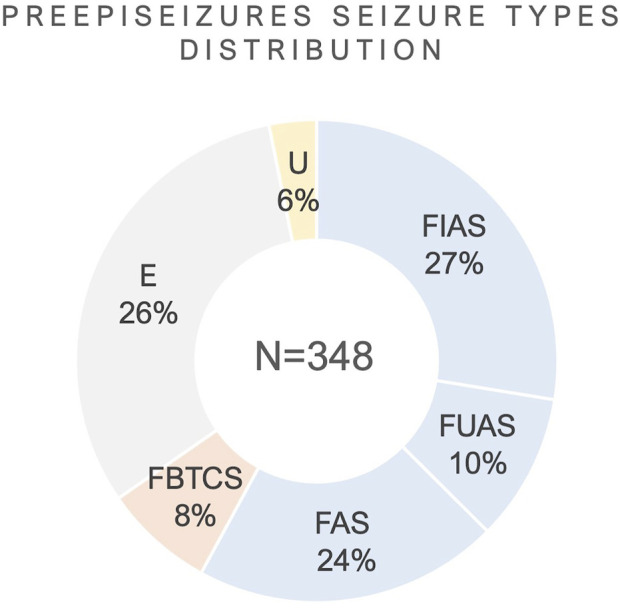
Distribution of seizure types from the cohort. *E*: Subclinical; *U*: Unknown; FIAS, Focal Impaired Awareness Seizure; FBTCS, Focal to Bilateral Tonic-Clonic Seizure; FAS, Focal Aware Seizure; FUAS, Focal Unknown Awareness Seizures.

The expansion of the data acquisition to another centre was possible due to the reproducibility of EpiBOX. Figure 5 shows the time domain analysis of wearable data recorded for all patients enrolled in this hospital. This set is composed of 15 patients, who performed the wearable acquisition with the EpiBOX setup, along with the ChestBIT device. From this group, one patient did not register any data during the acquisition, hence being excluded from this plot. The time domain analysis of the remaining 14 patients ranges from 10 min up to 3 days and 9 h. The average percentage of actual recorded time domain is 64.2%, with a standard deviation of 34.0%. Nonetheless, the median recorded time percentage rises to 83.6% (the median of time loss is only 16.4%). The total of seizures in this cohort is 45 clinical seizures (FIAS = 11, FAS = 34) and 20 subclinical seizures. As it is observed in Figures 5, 6 patients do not have reports on clinical seizures: 2 only experienced subclinical seizures; and 4 did not experienced any seizure during the exam. Nonetheless, the wearable device was able to capture 15 clinical seizures in this cohort.

Besides the data acquired in the scope of our project, the hospital also provided additional retrospective data, from other patients with temporal lobe epilepsy, who underwent the same monitoring exam. This set consisted on 9 patients, who experienced 61 clinical seizures (FBTCS = 1, FIAS = 36, FAS = 20 and FUAS = 4). This retrospective data contains the hospital system’s measurements, which includes the ECG. This cohort was used to initiate the ECG analysis and assess the validation of ECG-based seizure manifestations.

### 4.3 Overview

The patients collected in this database were performing the video-EEG monitoring for presurgical assessment. This section will detail the distribution of onset localisation and lateralisation, seizure types and ictal semiology reported by the clinical team.

In this cohort (59 patients), 64.41% (*N* = 38) showed temporal lobe activation during the onset of ictal period. The frontal lobe ictal onset activation showed a prevalence of 25.42% (*N* = 15), closely followed by the parietal lobe with 18.64% (*N* = 11). Lastly, the occipital lobe ictal onset was reported in 6.78% (*N* = 4). The ictal onset activation reported in more than one lobe region (e.g., fronto-temporal) was included in both categories. Concerning lateralisation, 38% had a left hemisphere onset seizure, whereas the epileptogenic zone was in the right hemisphere for 44% of patients. Bilateral ictal onsets were described in 4% of patients from the cohort.

In Figure 6, the distribution of seizures per seizure types is shown, regarding the seizure types which were reported in the video-EEG reports. The total number of seizures in the PreEpiSeizures dataset is 348, distributed a across six seizure types (FBTCS = 27, FIAS = 95, FUAS = 35, FAS = 82, subclinical = 89 and unknown = 20). These values are illustrated as a percentage in Figure 6, where the predominance of focal seizures is notable. The classification of seizures as Unknown was chosen when there was no information regarding the type of seizure, whereas FUAS correspond to focal seizures, in which only the awareness was not tested.

**FIGURE 7:**
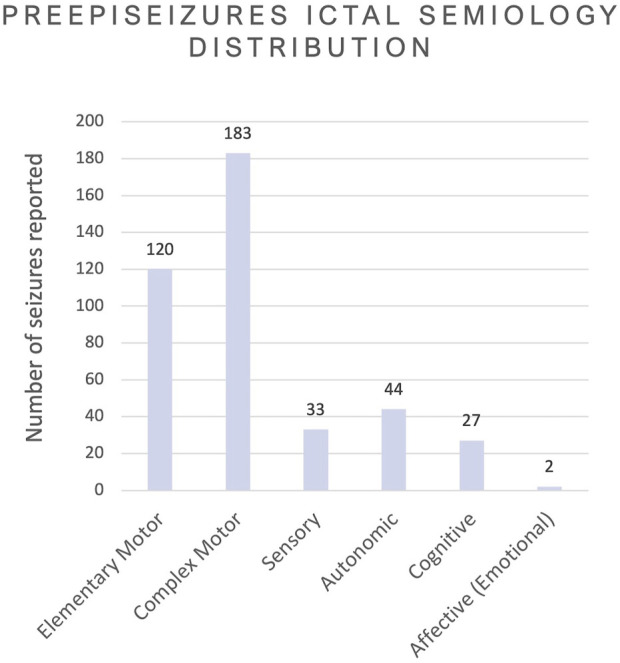
Overview of all ictal semiology phenomena reported in the video-EEG, grouped according to the nature of the phenomena following the 2022 ILAE’s glossary (Beniczky et al., 2022).

Besides the classification of seizure types, the video-EEG reports also contained some details regarding the events occurring during the seizure. In Figure 7, the reported ictal semiology from the dataset is displayed in 6 categories. The Elementary Motor Behaviour was found in 120 seizures and includes Dystonic (*N* = 23), head version or head orientation (*N* = 45), eye blinking (*N* = 12), myoclonic/clonic (*N* = 14), tonic (*N* = 6) and tonic-clonic (*N* = 27). The Complex Motor Behaviour (*N* = 183) included automatisms and hyperkinetic behaviour (*N* = 3). The most common automatisms were distal (*N* = 67) followed by oroalimentary (*N* = 59) and proximal (*N* = 16). Other reported automatisms were: verbal (*N* = 11); vocal (*N* = 6); mimic (*N* = 7); nose-wiping (*N* = 9) and Rinch (*N* = 5). Sensory phenomena (*N* = 33) was mostly populated by the somatosensory symptoms (*N* = 15), followed by cephalic aura (*N* = 5). Other reported sensory phenomena was gustatory (*N* = 1) and olfactory (*N* = 2). The most reported autonomic semiology was epigastric aura (*N* = 20), followed by hyperventilation (*N* = 12), tachycardia (*N* = 11) and piloerection (*N* = 1). Few seizures reported cognitive ictal phenomena (*N* = 27), where the most common symptom was autoscopy (*N* = 12), followed by dejá vu (*N* = 9), ictal aphasia (*N* = 3) and dysarthria (*N* = 3). Only 2 seizures reported emotional phenomena and the symptom was fear.

**FIGURE 8:**
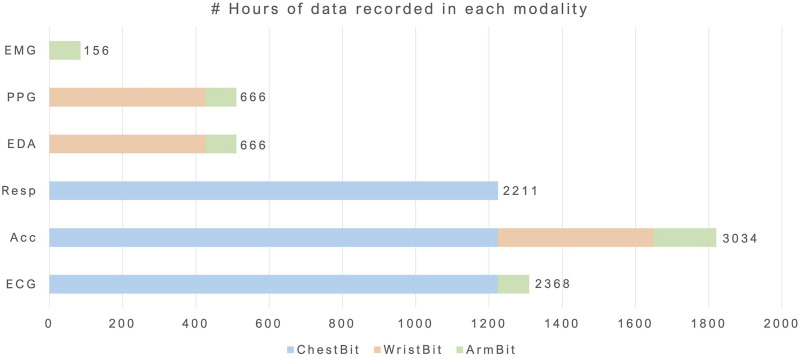
Number of hours acquired per modality. Each colour represents one form factor.

In Table 3, the total duration of ArmBIT, ChestBIT and WristBIT is shown separately. Figure 8 gives a visual depiction of the number of recorded hours by each modality. The different colours represent the device responsible for each measurement. The least represented modality is EMG with 156 h, 14 min and 54 s of data. Since this measurement required gel electrodes, it was discarded in the new devices. The EDA totals 666 h, 19 min and 2 s of data, which is decomposed in 156 h of using pre-gelled electrodes (with ForearmBIT) and 510 h, 4 min and 7 s of metallic electrodes in the WristBIT. The chest-ACC corresponds to 2,211 h of data, whereas the wrist-ACC totals 510 h. Additionally, the ArmBIT and ForearmBIT add more 312 h to the ACC data. Regarding RESP, 2,211 h were recorded so far, using only the ChestBIT. The ECG was captured using pre-gelled electrodes in 156 h (ArmBIT) and the ChestBIT has so far recorded 2,211 h. The total number of recorded hours by all wearables is 2,721 h, 57 min and 36 s. The broader use of ChestBIT instead of WristBIT was based on three arguments: an easier reproducibility; a higher reliability of ECG instead of PPG to record cardiac activity; and being more discreet, since it is placed under the clothes. This last point is aligned with previously surveyed preferences of PWE (Simblett et al., 2020).

To conclude the overview of the PreEpiSeizures dataset, the aforementioned Table 1 provides a side-by-side characterisation of this dataset with those available within the state-of-the-art. Three of the public databases (Bern-Barcelona (Andrzejak et al., 2012), New Delhi EEG (Swami et al., 2016) and Epilepsy Seizure Recognition (Andrzejak et al., 2001)) only presented segments of a few seconds duration. Bonn-Barcelona micro- and macro-EEG database (Martínez et al., 2020), comprises data from epileptic patients without epileptic seizures.

The comparison of our dataset with EPILEPSIAE (Klatt et al., 2012) and Siena Scalp EEG (Detti, 2020) is performed with regards to the ECG recorded by the hospital system (this is the number in the left side of columns patient and total hours). Our dataset size, both in terms of patients, and in total recorded hours, surpasses its peers.

Regarding wearable data, the only other dataset found with wearable data was the My Seizure Gauge Wearable, which does not contain ECG, PZT or Chest Motion data. Both datasets duration is quite similar (our wearable duration is the right number of column Total Hours in Table 1). Concerning the number of seizures and the variety of seizure types, PreEpiSeizures is similar to EPILEPSIAE and My Seizure Gauge Wearable. Regarding the number of recorded hours, the proposed dataset also closely follows those of the literature.

## 5 Discussion

The patients which are admitted for video-EEG monitoring are usually patients that either have refractory epilepsy and require a differential diagnosis, or are included in the presurgical evaluation program. In HSM, a new patient comes every 2 weeks, with some exceptions like vacations or other unforeseen events, which can change this schedule. Then, the patient stays for a work week (Monday to Friday) and sometimes also spends the weekend if the hospital team finds it necessary, for example, if no seizure occurred until Friday. Although this is a very good pace, allowing for up to 26 patients per year, not all these patients meet the eligibility criteria: patients need to have at least 16 years old; the EEG monitoring should be performed with scalp electrodes, instead of intracranial; patients also require a high level of independence, compatible with using an extra device continuously throughout the hospital stay. In HEM, the EEG technicians are all day with the patients (from 8AM to 11PM), hence the majority does not need accompaniment. The cadence in this ward is of 1–2 patients per week, however only one is asked to participate in the study. This is related also to the duration of the monitoring, since one patients stays in the hospital for the work week, whereas the other only stays for 48 h (the case of differential diagnosis) or less (in the case of other exams).

Despite having a great number of seizures, the proposed dataset suffers from one core issue also found in its peers: the great variability of seizure types. In both hospitals, many types of seizures are monitored (from tonic-clonic and myoclonic to subclinical seizures), and often the epileptogenic zone is not even known prior to the monitoring. As a result, it is difficult to predict what will be the outcome of the data collection. So far, the majority of seizures encountered have a focal onset, nevertheless they vary in duration, awareness or semiology. Even the broader classifications of seizure types (such as FAS and FIAS) enclose seizures that have motor and non-motor manifestations. Although patients in video-EEG monitoring have refractory epilepsy, their seizure occurrence is often highly reduced in daily life by ASM. During the monitoring, the ASM is slowly suspended to allow for resurgence of epileptic activity. For some patients, the complete withdraw of ASM could result in bilateral tonic-clonic seizures, which would hamper the identification of seizure onset. The goal in video-EEG monitoring is to achieve the balance that allows to clearly identify the ictal onset of seizures, with the minimum number of seizures. However, for some patients, no seizure occurs during the hospital admission, despite their frequent seizure rate outside the hospital.

The wearable data acquisition itself was an eventful process, filled with a great variety of challenges. Hereinafter, this section is divided into an overview of the problems faced and proposed solutions, followed by a discussion of the quality between the wearable device and the hospital system, and finally the quality assessment of the current acquisition setup and its comparison with the previous setup.

In Table 4, the various issues faced during the wearable data acquisition process are described, being divided into four categories according to their nature: real-world acquisition; wearable design; processing; and noise interference. Though this data collection was specific to a singular disease and a strict protocol, the challenges herein faced are general to wearable and even some non-wearable data collection experiments.

**TABLE 4:** Common concerns of wearable data acquisition.

Name	Description	Nature	Solution
Forget battery exchange	Uncontrolled scenarios can lead to forgetting battery exchanges	Real-world acquisition	Mobile app
User Compliance	The acceptance to participate and the proper realisation of the experiment	Real-world acquisition	Reward system
Incorrect placement	Uncontrolled scenarios can lead to incorrect device placement by non-experts	Real-world acquisition	Mobile app
Cohort’s variability	Real-world settings might translate into an unexpected variability to the cohort	Real-world acquisition	Multi-centre
Battery size	Battery shortage could cause more connection losses	Wearable design	Reduce FS
Design constraints	The signals to record are restricted by the sensors’ requirements	Wearable design	Adaptative factor
Device resistance	Resistance to sweat and to the continuous use over multiple acquisitions	Wearable design	Detachable electronics
Materials Hygienisation	Materials properly clean before and after each new acquisition, cleanable materials	Wearable design	Resistant materials
Signal quality	Uncontrolled scenarios can lead to noise presence in the signals of a variety of natures	Processing	Multimodal denoising
Temporal synchronisation	Signals acquired in different sources may present a relative temporal shift	Processing	HR synchronisation
Big data	High sampling frequencies and long hours leads to great amounts of data	Processing	Batch processing
Fragmented files	File fragmentation can lead to contiguous files with different signal offsets or amplitudes, sometimes time loss is unknown	Processing	Discrete analysis
Device drift	Devices can drift and originate signals with slightly different characteristics	Processing	Constant calibration
Motion artifacts	The presence of motion artifacts affects the proper measurements of other signals	Noise Interference	ACC denoising
Sensor interference	The continuous use of a device might degrade its sensors, and contamination can occur	Noise interference	Automatic check
Contact loss	Contact with the body can be lost during acquisition	Noise interference	Automatic check
Network interference	The surroundings can cause electrical disturbances in the physiological signal	Noise interference	Low-pass filtering
Baseline wander	Fluctuations on the ECG signal are common and might result in incorrect measurements	Noise interference	Baseline removal techniques

Four challenges were identified in relation to real-world acquisition: forget battery exchange (1.1); user compliance (1.2); incorrect placement (1.3) and cohort’s variability (1.4). Although all data was collected in the hospital ward, there was no constant supervision by an expert, leading to several challenges, that can also be expected in an ambulatory scenario (hence their “real-world” nature). (1.1) Most wearable devices require a quasi-daily change of battery, as seen in Table 2. Hence, it could be expected that users forget to charge the device or to have a charged battery at-hand. To help in this process, the proposed solution is a mobile application with reminders to charge the device or change the battery. (1.2) User compliance is important for any experiment, especially those in which the patient plays a major role in the acquisition control. Mental and emotional availability is required to perform a rigorous experiment control; despite the relative good rate of potential patients, not all will meet the inclusion criteria to participate in the data collection. Moreover, this is amplified in ambulatory data collection, and strategies should be established to increase patient’s compliance. As a suggestion, a more immediate reward system could be added to the protocol, such as their data overview, or daily statistics. (1.3) Unsupervised experiments such as this, could lead to incorrect placement of the devices, especially if more than one device is used simultaneously. For example, the ChestBIT could be inverted from its original orientation, or shifted to one side or the other. Moreover, the ChestBIT could fall during the night or even detach itself. In order to solve these issues, the device should be reinforced especially in the attachment area. Moreover, in an ambulatory setting, the mobile application could aid the patient to properly adjust the device, for example, via a signal quality assessment algorithm. (1.4) The variability of the cohort is something to account for when dealing with such specific diseases. Potential patients could have different ages, unique disease characteristics, or even specific comorbidities. In this case it could be useful to design the protocol upfront as a multi-centre data collection. The increase of potential patients could enable the encounter of similar cases and better deal with the great cohort’s variability.

The category of wearable design included four challenges: battery size (2.1); design constraints (2.2); device’s resistance (2.3); and materials hygienisation (2.4). The nature of these challenges comprises issues which conditioned the wearable design, and should be accounted for when proposing new designs for continuous wearable data collection. (2.1) The battery size is a constraint on wearable devices, especially in preliminary versions, based on lithium ion polymer batteries, hence their energy consumption should be reduced to the minimum, to ensure the maximum operability. In the proposed devices, the FS is 1,000 Hz, which corresponds to a battery drain rate of approximately 62 mAh (i.e., milliamp hour). As a result, 1,500 mAh are required for lasting 24 h. One solution could be to decrease the FS to 100 Hz for the ChestBIT (or less in the case of the WristBIT), to enable a slower rate of battery drain, while still capturing the major physiological manifestations. (2.2) The design of a wearable device is conditioned to its embedded sensors. For example, the ECG sensor requires at least two contact points with the body, one in each side of the heart, whereas the EDA has better quality in places with more eccrine glands (Nagai et al., 2019). Thus, when designing a new wearable it is necessary to understand which modalities are more relevant to the task, since not all are possible to acquire with the same form factor. (2.3) The device should be resistant to ensure long-term use without quality loss. This could be achieved by choosing appropriate materials, by attaching securely the electronics to the textiles, or even by adopting bendable electronics. (2.4) The materials disinfection is important to remove bacteria and fluids, especially during patient exchange. The choice of materials could also play an important part, not only comfort-wise but also for proper hygienisation.

The next category of challenges is processing, and it includes: signal quality (3.1); temporal synchronisation (3.2); big data (3.3), fragmented files (3.4); and device drift (3.5). Processing concerns the first steps towards the extraction of meaningful information from the acquired data. The challenges herein described could be detrimental and lead to misinformation, thus they should be carefully addressed, preferably during the actual data collection. (3.1) As it was previously mentioned, the device could be incorrectly placed due to various reasons. Moreover, other factors (which will be addressed in the next category) could contribute to poor quality signals. Quality control should be assured automatically through an algorithm, specific for each modality acquired. (3.2) When acquiring data from more than one device simultaneously, or when comparing two signals acquired simultaneously (such as the hospital’s ECG against the device’s ECG), or even when using the hospital’s annotations with the wearables, it is relevant to confirm whether the temporal line is properly defined. It could be the case that the wearable does not save data with the correct time. To solve this issue, it should be implemented a temporal synchronisation. The temporal synchronisation between the same modality time series is possible using cross-correlation (e.g., in ECG/PPG, one can use the instantaneous heart rate from both sources). This could be used between the hospital’s signal and the device’s signal, and the devices time should be adjusted according to this temporal offset. In our case the EpiBOX smartphone’s application allows to save the device’s time alongside the real time (fetched by the smartphone). This time difference could also be used to adjust the time to a better estimation prior to signal synchronisation.

(3.3) The FS of 1,000 Hz, associated to several days of acquisition, originates several gigabytes of data per patient. This poses a problem for opening such files during the processing phase. One solution could be batch processing; through this technique, the time is divided into intervals of a few hours (or even less than 1 h), and the signals are extracted from the TXT format to a more compressed format, such as parquet (a columnar file optimized for processing big data). This could allow to save space and time in future operations. (3.4) There is always the case of ending one file and starting another in the middle of the acquisition. This also occurs for hospital files. While it is expected that the next file will be in the same conditions as the previous, changes can occur in both signal’s amplitude and/or offset. Moreover, battery exchange can lead to a slight change in the devices position. During processing, the timeseries should be considered discontiguous if they come from fragmented files, and processing should occur separately for each fragment. (3.5) As it was previously mentioned, a drift in device position during the course of the acquisition is inevitable, due to several reasons. The timeseries should be seen as an evolving signal, and not static according to an initial defined baseline. The normalisation or even signal shape correctedness should be assessed with nearby points and not the entire signal. This does not invalidate standard references of quality for the entire signal, but simply whether signal changes are a result of drifts or they contain meaningful information towards the final purpose.

Noise interference in timeseries is especially prominent in wearable acquisitions, with a multitude of noise types (Vijayan et al., 2021). Noises can be difficult to remove when they fall into the frequency bands of the acquired signal. The following noises found in the data acquisition were: motion artifacts (4.1); sensor interference (4.2); contact loss (4.3); network interference (4.4); and baseline wander (4.5). The proper identification of each noise type, could be the first step for their automatic removal during the processing phase. (4.1) Motion artifacts fall into most signals frequencies of interest, thus their removal is not trivial. Since acceleration is acquired in most wearable devices, one solution could be to use the ACC signal to remove motion from the other signal modalities. This would mostly work when the sensors are placed in proximity. For example, the ACC from WristBIT could have common motion with the EDA, however motion captured in the PPG acquired at the finger might not have been captured by the ACC. (4.2) The continuous long-term use of the device could deteriorate the electronic board contacts and originate improper signals, which should be tested regularly. (4.3) Loss of contact between the device and the skin is something to account for, since the connection is still maintained but the desired signal is no longer there. This should also be assessed automatically by identifying the types of signal shapes induced by such issue, and seeking them in the original signal prior to meaningful extraction. (4.4) The presence of other electronic devices in the surrounding space can influence the quantity of noise in the signal. The video-EEG room contains a great amount of electrical devices acquiring simultaneously and constantly, and one should be prepared for a small signal-to-noise ratio. (4.5) The presence of baseline wander, especially in the ECG signal, is common in both the wearable device and the hospital’s system. Similarly to motion artifacts, baseline wander can also fall in the signal frequency range and affect meaningful information extraction. There are several baseline wander techniques proposed in the literature which could be implemented during the processing step.

Given this last set of challenges, one potentially valuable addition to the PreEpiSeizures dataset would be to provide the quality assessment within the metadata of each file. The developed approach should be based on the assessment of quality against the physiological basis in an agnostic manner. This will be addressed in the future work.

## 6 Conclusion

The PreEpiSeizures project was motivated by the limited number of available wearable data in epilepsy; this collaboration has enabled the collection of data from 59 patients over the past few years (where 37 patients also wore a chest device, nine wore a wrist device, and four wore an arm device). The data collection resulted in 5,838 h of hospital data and 2,721 h of wearable data.

So far, three different form factors of wearable devices were worn by the patients: ArmBIT, WristBIT and ChestBIT. The ChestBIT’s ability to record the ECG signal using non-gelled conductive materials, is an advantage compared with the ArmBIT. The quality assessment of ChestBIT was at the level of the hospital system (in the case of the ECG), and WristBIT quality was comparable to the quality of Empatica E4 used in similar conditions, reported in previous works. The advantage of using our own devices, allowed to have the liberty to experiment with form-factors and physiological modalities. Although not all patients have used the wearable devices, the availability of the hospital’s ECG signal allows to draw similar conclusions regarding the physiological manifestations of epilepsy in cardiac activity, which is also aligned with the literature.

The video-EEG monitoring in the hospital significantly reduces the variability of the real-world while allowing the access of ground truth annotations. The ambulatory setting may reveal additional challenges which are not being considered yet, nevertheless it was possible to identify 18 challenges that impacted the wearable data collection and may also appear in real-world scenarios. Further research on these challenges should be a priority to avoid data and time losses. Moreover, the concerns and challenges raised in the discussion affect overall continuous health monitoring studies and are not exclusively epilepsy monitoring.

The future of the PreEpiSeizures project will include the maintenance of the data collection process, as well as the curation of the dataset and the expansion to ambulatory monitoring is also envisioned. Additionally, the project will address some of the acquisition challenges mentioned, such as: the battery size, the real-time signal quality assessment, and noise reduction.

## Data Availability

The raw data supporting the conclusion of this article will be made available by the authors, without undue reservation.

## Footnotes

1
https://www.ieeg.org/

2
https://eithealth.eu/spotlight-story/seizeit/

3
https://www.epilepsyecosystem.org/

4
https://www.epilepsyecosystem.org/my-seizure-gauge-1

5
https://www.empatica.com/en-eu/research/e4/

6
https://www.polar.com/us-en/products/accessories/polar-soft-strap

7
https://us.nihonkohden.com/

8
https://micromedgroup.com/
