# Supplementary material for: PreEpiSeizures: description and outcomes of physiological data acquisition using wearable devices during video-EEG monitoring in people with epilepsy
Source: Front Physiol. 2023 Oct 10;14:1248899. doi: 10.3389/fphys.2023.1248899 (PMC10597694; doi:10.3389/fphys.2023.1248899)
Supplement: Supplementary file 1 [file Presentation1.pdf]

frontiers

Supplementary Material

1 FILE HEADER LIST

The following list describes the most relevant entries of the header, with some examples:

- sensor - a list of the names of the sensors being used (ex: [ECG, PZT , ACCx, ACCy, ACCz, -])
- device name (ex: ”Device 1”)
- column - a list mapping the columns to the analogue channels (ex: [NSeq, A1, A2, A3, A4, A5, A6])
- time - starting time of the file with a high resolution - H:M:S.MS (ex: 18:09:05.923879)
- device connection - the Mac-Address, which is the device’s ID (ex: 5C:02:72:9F:53:A0)
- channels - the index of each column which contains sensor data (ex: [1, 2 ,3, 4, 5, 6])
- date - the starting date in the format Y-M-D (ex: 2021-04-02)
- sampling rate - the FS being used (ex: 1000)
- label - the label to give to each sensor column (ex: [ECG, resp, ACCx, ACCy, ACCz, raw])
- resolution - the resolution with each sensor was recorded (ex: [10, 10, 10, 10, 6, 6]).
